# Surgical technique for the successful curative resection of locally advanced caecal cancer invading the external iliac artery: A case report

**DOI:** 10.1016/j.ijscr.2021.106550

**Published:** 2021-11-03

**Authors:** Akira Kubota, Toshiyuki Yamazaki, Hitoshi Kameyama, Hideki Hashidate, Tomoyuki Imai, Takashi Wakabayashi

**Affiliations:** a Department of Digestive Surgery, Niigata City General Hospital, 463-7 Shumoku, Chuo-ku, Niigata City, Niigata 950-1197, Japan; b Department of Pathology, Niigata City General Hospital, 463-7 Shumoku, Chuo-ku, Niigata City, Niigata 950-1197, Japan; c Department of Urology, Niigata City General Hospital, 463-7 Shumoku, Chuo-ku, Niigata City, Niigata 950-1197, Japan; d Department of Cardiovascular Surgery, Niigata City General Hospital, 463-7 Shumoku, Chuo-ku, Niigata City, Niigata 950-1197, Japan

**Keywords:** Vascular reconstruction, Curative resection, Advanced cecal cancer, Femoral-femoral bypass, Neoadjuvant chemotherapy, Case report

## Abstract

**Introduction:**

Curative resection generally has a good prognosis if the tumor is a locally advanced colorectal tumor. However, resection of a primary tumor that has invaded the aortoiliac artery is controversial. Herein, we report a case of successful resection of advanced cecal cancer invading the external iliac artery.

**Case report:**

A 29-year-old male patient had advanced cecal cancer invading the right external iliac artery and vein, right ureter, iliopsoas muscle, and sigmoid colon. We collected the patient's pre-/intra-/postoperative, clinical, and histological data. We reviewed the factors that may have contributed to curative resection without complications. We performed a palliative terminal ileum-sigmoid anastomosis for the prevention of intestinal obstruction. The patient received neoadjuvant chemotherapy, and the tumor patently regressed. After arterial reconstruction was performed with a femoral-femoral bypass, we performed radical resection: right hemicolectomy; partial sigmoidectomy; and partial resection of the right ureter, iliopsoas muscle, right testicular, and external iliac vessels. Pathologically, 99% of the tumor cells disappeared after chemotherapy. The patient was discharged on postoperative day 9. No recurrence has been noted 24 months after surgical resection, and the patient is receiving adjuvant chemotherapy.

**Conclusions:**

Thus, we successfully resected advanced cecal cancer without complications. Reconstruction with femoral-femoral arterial bypass and neoadjuvant chemotherapy are useful methods for curative resection without complications.

## Highlights

• Our patient had advanced caecal cancer invading the external iliac artery.
• We performed reconstruction with femoral-femoral arterial bypass.
• The patient underwent neoadjuvant chemotherapy.
• We successfully resected the advanced caecal cancer without complications.

## 1 Introduction

Colon cancer with aortoiliac invasion is generally unresectable because patients have a high risk of morbidity and mortality, and curative resection is difficult [1]. However, it has been reported that patients who have undergone curative resection have a good prognosis, even if the tumor is locally advanced [2], [3], [4], [5]. Although vascular reconstruction has been performed previously in the process of radical resection, cases of primary tumor resection with vascular reconstruction have not been reported. The reported cases are those of local or lymph node recurrence but did not include primary tumor invasion [6], [7], [8]. To the best of our knowledge, this is the first report of successful primary tumor resection with simultaneous digestive and vascular reconstruction. In this report, we present the case and review the relevant literature regarding contributing factors toward curative resection without complications.

## 2 Patient information

**Fig. 1:**
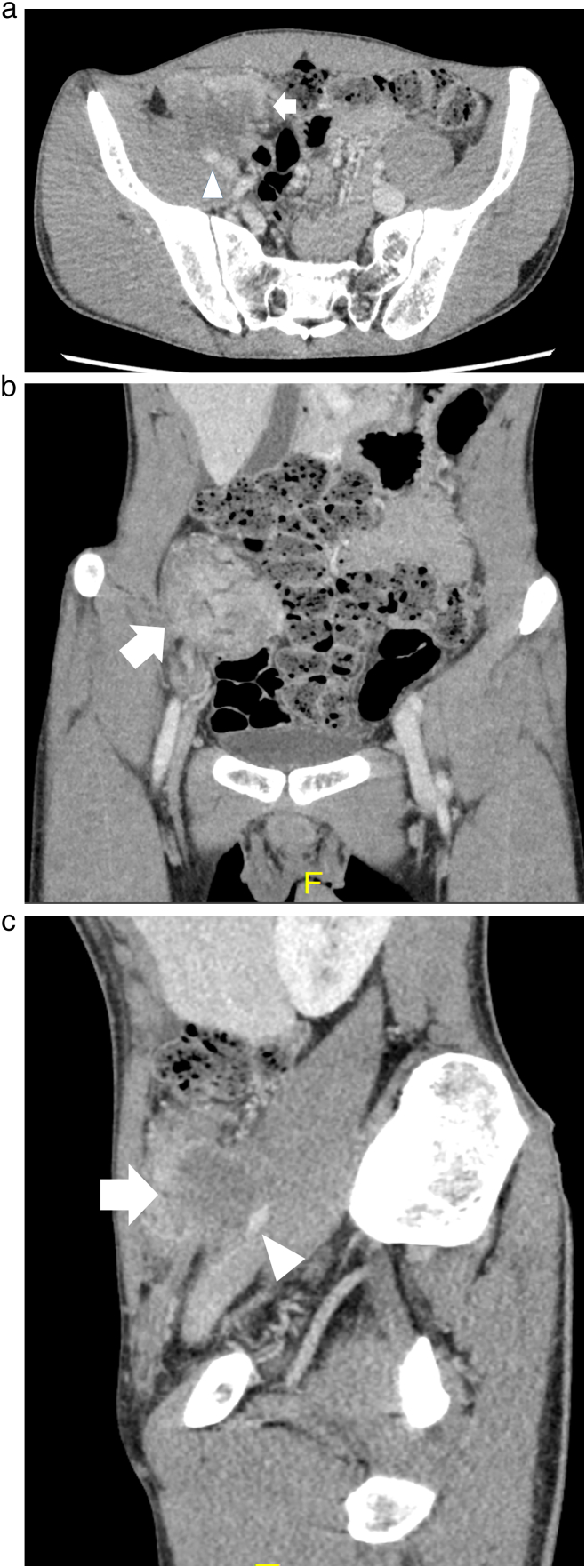
(a) Computed tomography (CT) findings at the first visit (axial). The cecal tumor invading the external iliac artery (arrow) and stenosis of the external iliac artery (arrowhead). (b) CT findings at the first visit (coronal). The cecal tumor (arrow). (c) CT findings at the first visit (sagittal). The cecal tumor invading the external iliac artery (arrow) and stenosis of the external iliac artery (arrowhead).

A 29-year-old male patient with lower right abdominal pain was referred to our hospital. The patient had no family history of colon cancer. A cecal tumor was found via computed tomography (CT). Furthermore, the tumor had invaded several organs: the right external iliac vessels, right ureter, iliopsoas muscle, and sigmoid colon (Fig. 1a–c). Although we performed a diagnostic colonoscopy, we could not reach the tumor and could not perform the biopsy. Although there was no occlusion, we planned for primary tumor resection following surgical biopsy of the tumor, digestive bypass, and neoadjuvant chemotherapy.

**Fig. 2:**
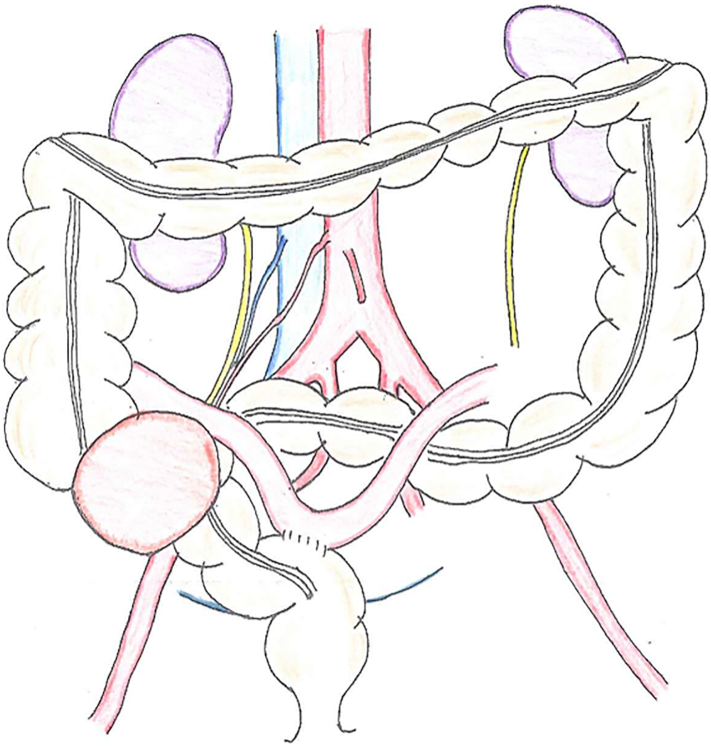
Illustrated palliative bypass of the small intestine-sigmoid colon.

**Fig. 3:**
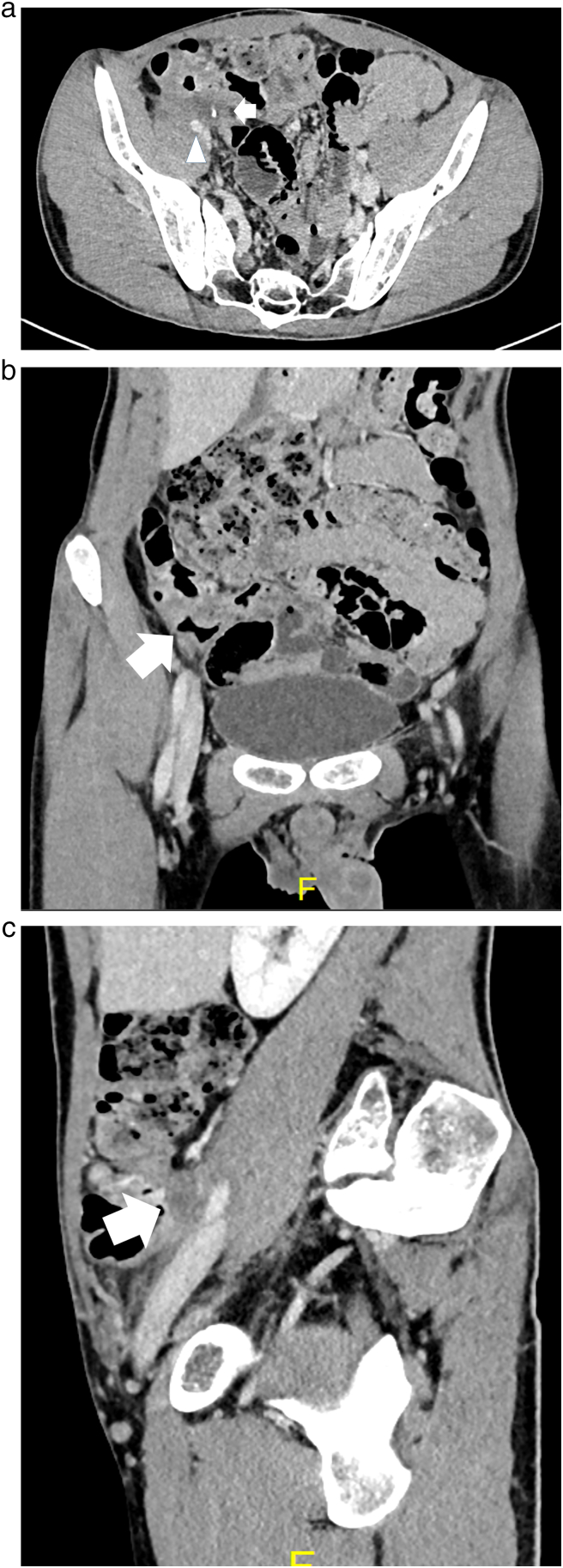
(a) Computed tomography (CT) findings following chemotherapy (axial). Regression of primary tumor (arrow) and improvement of external iliac arterial stenosis (arrowhead). (b) CT findings following chemotherapy (coronal). Regression of primary tumor (arrow). (c) CT findings following chemotherapy (sagittal). Regression of primary tumor (arrow).

The patient underwent a surgical biopsy of the lymph node near the tumor and a palliative terminal ileum-sigmoid anastomosis to the anal side from the tumor invaded position for the prevention of intestinal obstruction (Fig. 2). The tumor was diagnosed as an adenocarcinoma. The patient received six courses of infusional fluorouracil, leucovorin, oxaliplatin, and irinotecan (FOLFOXIRI) plus bevacizumab for 2–5 courses as neoadjuvant chemotherapy. The tumor regression rate was >60%, as measured on CT, and the patient had a partial response (Fig. 3a–c) [9]. Furthermore, the serum carbohydrate antigen 19-9 (CA19-9) level, which was 185.4 U/mL at diagnosis, decreased to 10.8 U/mL. Serum carcinoembryonic antigen (CEA) levels were within the normal range. There was no distant metastasis seen on CT, and we decided to perform the surgery.

## 3 Surgical findings

**Fig. 4:**
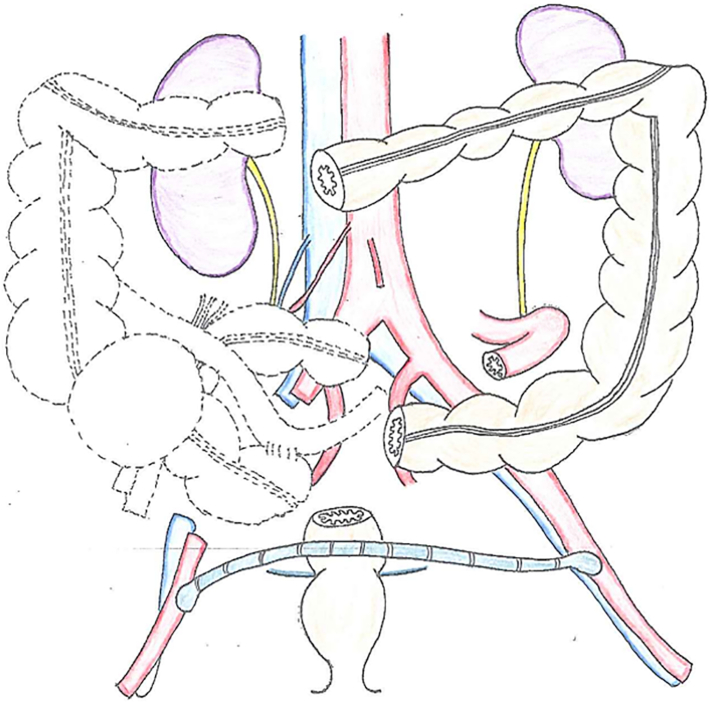
Illustrated following radical resection.

**Fig. 5:**
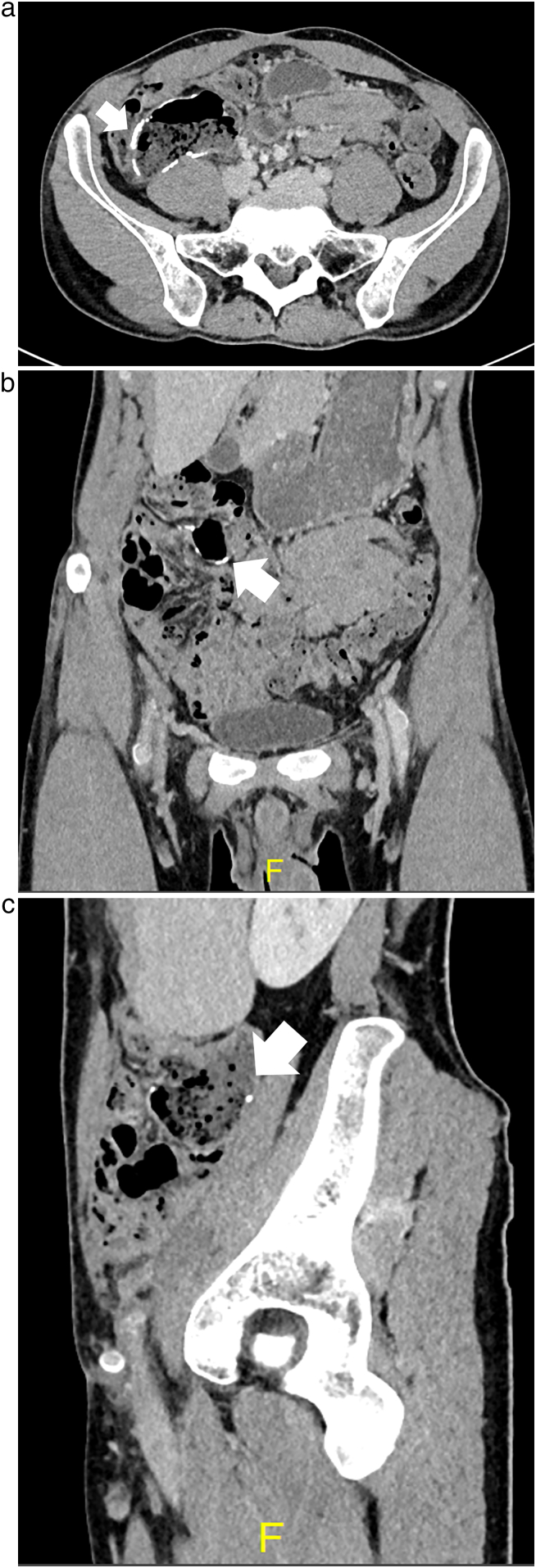
(a) Computed tomography (CT) findings following resection (axial). Anastomosis (arrow). (b) CT findings following resection (coronal). Anastomosis (arrow). (c) CT findings following resection (sagittal). Anastomosis (arrow).

The operation was conjointly performed by colorectal, urology, and cardiovascular surgeons. First, the urology surgeon inserted a stent in the right ureter to preserve the ureter. Subsequently, a cardiovascular surgeon performed a femoral-femoral arterial bypass. The bilateral inguinal incisions were protected by wound dressing to prevent wound infection. We performed laparotomy and right hemicolectomy, partial sigmoidectomy, and partial resection of the right ureter, iliopsoas muscle, right testicular, and external iliac vessels (Fig. 4, Fig. 5a, b, c, 6a, b). Reconstruction of the intestinal tract was performed by functional end-to-end anastomosis for the ileum-transverse colon and DST for the descending colon and rectum.

**Fig. 6:**
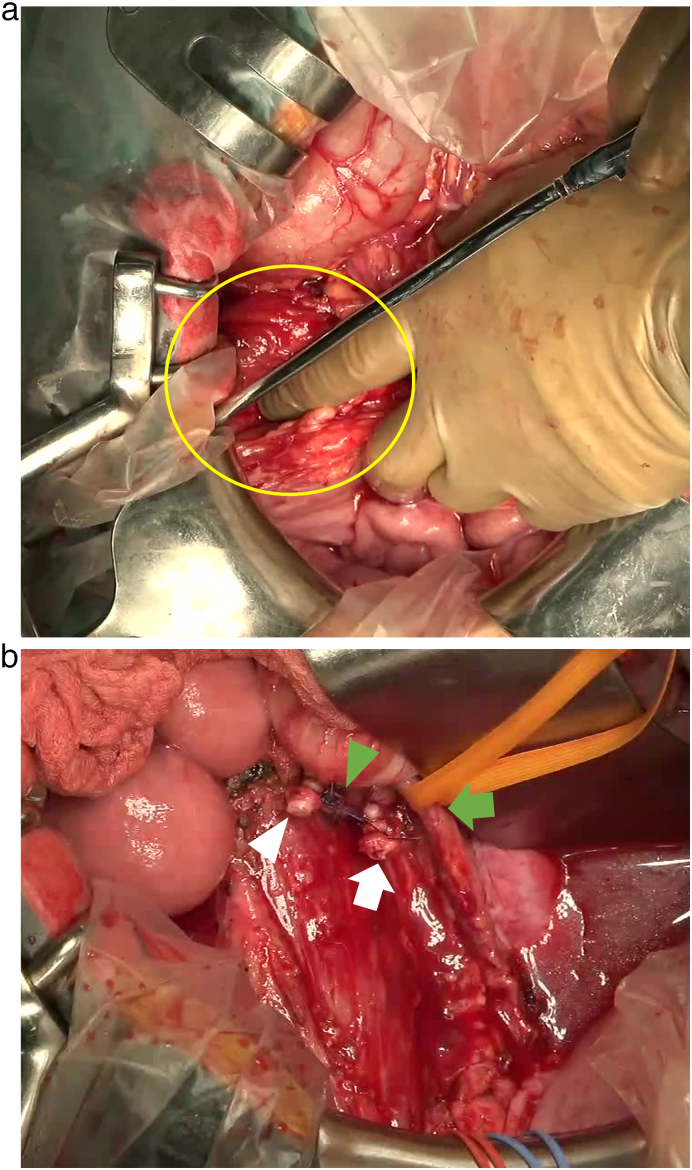
(a) Photo of the lesion before resection. (b) Photo of the lesion following resection.

Although we did not reconstruct the right external iliac vein and ureter in consultation with the cardiovascular and urology surgeons, there was no edema of the right lower limb or renal dysfunction, and the kidney spontaneously regressed. Overall, 590 mL of blood was loss, and the operative time was 530 min.

## 4 Histopathological findings

**Fig. 7:**
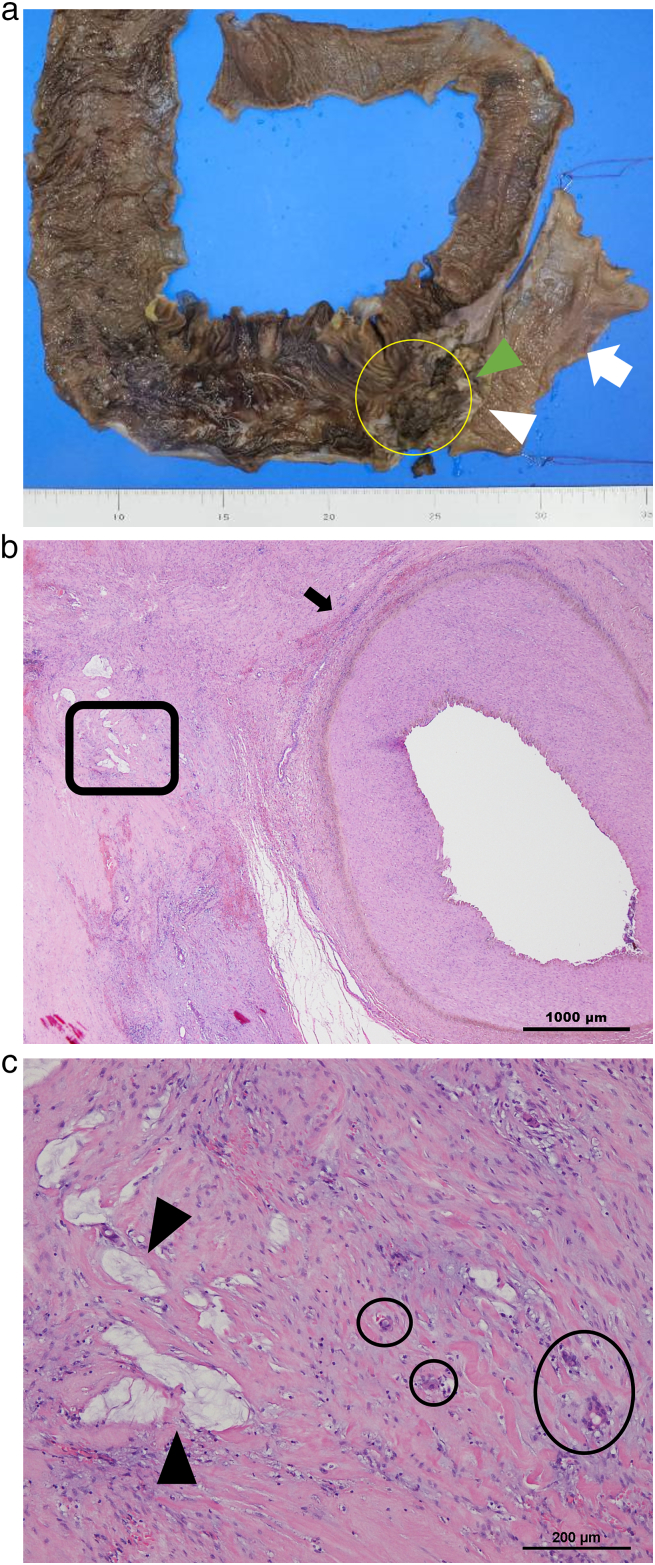
Pathological findings. (a) A gross photo of the tumor after resection. (b) Fibrous tissue around the external iliac artery (arrow). (c) Magnification of the circled site in Fig. 4a. Mucus (arrowhead), and viable tumor cells (circles).

Histopathological examination showed that the response to chemotherapy was grade 2 [10], more than 99% of tumor cells had disappeared, and there was only a small number of tumor cells in the submucosa, muscularis propria, and soft tissue near the external iliac artery (Fig. 7a–c). KRAS and BRAF mutations were not detected. The invasive soft tissue around the right external iliac artery developed fibrotic scarring and calcification. The excision margin was pathologically negative.

## 5 Postoperative course

The patient was discharged on postoperative day 9 without complications and was able to perform recreational rock-climbing immediately, without any recovery time. The patient received six courses of infusional fluorouracil, leucovorin, and oxaliplatin (FOLFOX) 6 courses as adjuvant chemotherapy. No recurrence has been noted 24 months after surgical resection. We described the case according to the SCARE guidelines 2020 [11].

## 6 Discussion

Digestive and cardiovascular surgeries, which we performed simultaneously, have a high risk of surgical site infection (SSI). Previous studies report that SSI rates could be as high as 30% in patients undergoing colorectal surgery, thus justifying our decision to reconstruct the digestive tract [12]. In a study involving 12 patients with recurrent colorectal cancer with aortoiliac invasion, the complication rate was 75% (9/12) and SSI rate was 33% (4/12) [7]. We considered the risk of SSI to be even higher for primary tumor resection with digestive reconstruction than for recurrent tumor resection. To avoid the risk of SSI, we separated the surgical field by performing a femoral-femoral bypass. Revascularization of the external iliac artery must be performed intraperitoneally, but in the case of F-F bypass, revascularization is possible on the caudal side of the inguinal ligament. Femoral-femoral arterial bypass can reduce the risk of SSI and vascular graft infections due to separation of the surgical field into the intraperitoneal and inguinal regions. Notably, vascular graft infection is also associated with high mortality rates [13].

Neoadjuvant chemotherapy plays an essential role in the success of curative resection. In our present case, histopathological examination showed that more than 99% of the tumor cells had disappeared; there was only a small number of tumor cells in the submucosa, muscularis propria, and soft tissue near the external iliac artery. The invasive soft tissue of the primary tumor was scarred by fibrosis and calcification. Previous studies also report that neoadjuvant chemotherapy is a valid option for curative resection because of tumor regression [14], [15], [16]. A previous study showed that 35% of patients who had an unresectable tumor and received neoadjuvant chemotherapy had a curative resection, and the survival of patients receiving neoadjuvant chemotherapy was longer than that of those receiving primary tumor resection [17].

We believe that the reduced operative time due to omission of the right ureter and iliac vein reconstruction contributed to the lack of complications in this case. Although we did not reconstruct the right external iliac vein, the patient had no edema of the right lower limb. A previous study showed that in patients who underwent pancreatoduodenectomy with portal vein resection, the external iliac vein was used for reconstruction, they did not undergo reconstruction of the external iliac vein [18]. According to a follow-up CT, the right internal iliac vein system had developed, and the blood in the right external iliac vein had drained. Therefore, it may not be necessary to reconstruct the external iliac vein if the internal iliac vein can be preserved. Although we did not reconstruct the ureter, the patient had no renal dysfunction according to laboratory data.

The patient was discharged without complications and is receiving adjuvant chemotherapy to date. We believe that the use of femoral-femoral bypass surgery, reduction of blood loss, shorter operative time, and patient's good response to neoadjuvant chemotherapy were all contributing factors. The overall good health of the patient, who was young and active, could also be associated with the lack of complications. Previous studies have reported that the morbidity and mortality rates of invasive colorectal cancer resection are as high as 60% and 5%, respectively [16]. Radical resection with aortoiliac reconstruction is an additional high-risk factor for morbidity and mortality. Therefore, we must carefully assess the presence of distant metastasis, and patients with poor health status should not undergo surgery.

## 7 Conclusion

We successfully resected the locally advanced cecal cancer that invaded the external iliac artery. Reconstruction of femoral-femoral arterial bypass and neoadjuvant chemotherapy are useful methods for curative resection without complications.

## Ethics approval

This study was approved by the Ethics Committee of Niigata City General Hospital.

## Funding

This research did not receive any specific grant from funding agencies in the public, commercial, or not-for-profit sectors.

## CRediT authorship contribution statement

Dr. Toshiyuki Yamazaki was the first assistant surgeon. Dr. Hiroaki Uehara performed an intestinal bypass and surgical biopsy of the lymph node near the primary tumor. Dr. Hideki Hashidate made the pathological diagnosis. Dr. Takashi Wakabayashi and Dr. Akihiro Nakamura made a femoral-femoral arterial bypass. Dr. Tomoyuki Imai inserted a stent in the right ureter. Dr. Hitoshi Kameyama and Akira Iwaya revised the manuscript. All authors read and approved the final manuscript.

## Guarantor

Akira Kubota.

## Registration of research studies

N/A.

## Consent to participate

Written informed consent was obtained from the patient for his participation in this study.

## Consent for publication

Written informed consent was obtained from the patient for the publication of this case report.

## Availability of data and material

All data generated or analyzed during this study are included in this published article and its supplementary information files.

## Declaration of competing interest

The authors declare that they have no conflicts of interest.
